# Serological Cross-Reaction between Six Thiadiazine by Indirect ELISA Test and Their Antimicrobial Activity

**DOI:** 10.3390/mps6020037

**Published:** 2023-04-03

**Authors:** Mishell Ortiz, Hortensia Rodríguez, Elisabetta Lucci, Julieta Coro, Beatriz Pernía, Abigail Montero-Calderon, Francisco Javier Tingo-Jácome, Leslie Espinoza, Lilian M. Spencer

**Affiliations:** 1 School of Biological Sciences and Engineering, Yachay Tech University, San Miguel de Urcuquí 100119, Ecuador; 2 School of Chemical Sciences and Engineering, Yachay Tech University, San Miguel de Urcuquí 100119, Ecuador; 3 Departamento de Tecnología de Procesos Biológicos y Bioquímicos, Universidad Simón Bolívar, Caracas 89000, Venezuela; 4 Laboratory of Chemical and Biomolecular Synthesis, Faculty of Chemistry, Habana University, Habana 10400, Cuba; 5 Faculty of Natural Sciences, University of Guayaquil, Av. Raúl Gómez Lince s/n y Av. Juan Tanca Marengo, Guayaquil 090150, Ecuador; 6 School of Agricultural and Agro-Industrial Sciences, Yachay Tech University, San Miguel de Urcuquí 100119, Ecuador; 7 Biology Center, Central University of Ecuador, Quito 170129, Ecuador; 8 Cell Biology Department, Simón Bolívar University, Valle de Sartenejas, Caracas 89000, Venezuela

**Keywords:** malaria, thiadiazine, indirect ELISA, cross-reaction, antimicrobial activity

## Abstract

Malaria is a parasitic infection caused by a protozoon of the genus *Plasmodium*, transmitted to humans by female biting mosquitoes of the genus *Anopheles*. Chloroquine and its derivates have caused the parasite to develop drug resistance in endemic areas. For this reason, new anti-malarial drugs as treatments are crucial. This work aimed to evaluate the humoral response. with hyper-immune sera, of mice immunized with six derivatives of tetrahydro-(2H)-1,3,5-thiadiazine-2-thione (bis-THTT) by indirect ELISA test. The cross-reactivity between the compounds as antigens and their microbial activity on Gram-positive and Gram-negative bacteria was evaluated. The results of the humoral evaluation by indirect ELISA show that three bis-THTTs react with almost all of the above. Besides, three compounds used as antigens stimulate the BALB/c mice’s immune system. The best combination of two antigens as a combined therapy displays similar absorbances between the antigens in the mixture, showing similar recognition by antibodies and their compounds. In addition, our results showed that different bis-THTT presented antimicrobial activity on Gram-positive bacteria, mainly on *Staphylococcus aureus* strains, and no inhibitory activity was observed on the Gram-negative bacteria tested.

## 1. Introduction

The bite of female Anopheles mosquitos transmits Malaria when they ingest blood and introduce the *Plasmodium* parasite. This parasite is a protozoan of the phylum Apicomplexa [1]. The genus *Plasmodium* comprises five species that infect humans: *Plasmodium vivax*, *P. ovale*, *P. malarie*, *P. falciparum*, and *P. knowlesi*. According to the World Health Organization (WHO), malaria cases worldwide decreased from 238 million in 2000 to 230 million cases in 2019, meaning malaria still constitutes a global health problem. Up to 2020, WHO report that 77% of malaria deaths occur among the most vulnerable members of society. These are children under five and pregnant women whose exposure to this parasite causes prematurity, low birth weight, and even infant mortality [2,3,4]. The most frequent drugs used for Malaria are chloroquine and artemisinin combination therapy (ACT). 

Anti-malarial drugs have lost their effectiveness due to poor administration and prolonged use, leading to drug resistance development. The main causes of anti-malarial drug resistance are incorrect dosing, drug absorption, unusual pharmacokinetics, parasite mutation, cross-resistance, vector and environment, host immunity, and low transmission of Malaria. Another cause of resistance is treatment failure, such as trying to resolve clinical symptoms, despite using correct doses of the anti-malarial drug [5,6,7]. The spread of drug resistance on a large scale is a problem in the fight against the disease and has spurned research related to the creation of a new vaccine that can limit transmission [8,9].

The resistance generated by *Plasmodium falciparum* against Chloroquine is due to a mutation called PfCRT (Plasmodium falciparum chloroquine resistance) that occurs at position 76 (K76T) [8]. This mechanism is due to the parasite’s resistant strains against Chloroquine having a neutral threonine residue, instead of the PfCRT protein’s positively charged lysine moiety [10]. Artemisinin resistance is caused by the Kelch protein, specifically by point mutations in the phosphatidylinositol-3-kinase gene (Pfkelch13 or K13) of *P. falciparum* [8]. The mutation in K13 has been associated with the endoplasmic reticulum of the parasite and improper protein folding [11].

In previous studies, six tetrahydro-(2H)-1,3,5-thiadiazine-2-thione (THTT) derivatives were evaluated in vivo with two strains of rodent malaria, and some showed several antimalaria activities in a rodent model with *P. yoelii* and *P. berghei* [12,13,14]. 

According to IUPAC, heterocyclic compounds are “cyclic compounds having as ring members atoms of at least two different elements” [15]. Heterocyclic compounds have shown anti-bacterial, anti-viral, anti-fungal, anti-inflammatory, and anti-tumor activities. The heterocycles based on THTTs (tetrahydro-2H-1,3,5-thiadiazine-2-thiones) have anti-protozoal, anti-bacterial, anti-fungal, anthelmintic, and tuberculostatic properties because of their high lipid solubility, enzymatic rate of hydrolysis, and stability in a simulated gastric fluid that facilitates stomach absorption [16,17].

Herein, the performance of an indirect ELISA for cross-reactivity evaluation of six bis-THTTs (JH1, JH2, JH3, JH4, JH5 and JH6), previously prepared and characterized, was carried out to determine analysis by antibody recognition and to find alternatives to combined therapy via the combination of two antigens. In consequence, the indirect ELISA test allowed us to evaluate cross-reactivity for structurally similar organic compounds derived from bis-Thiadizines. Additionally, the antimicrobial activity on Gram-positive and Gram-negative bacteria of the THTTs derivatives (JH1 to JH6) was also determined.

## 2. Materials and Methods

### 2.1. Tetrahydro-(2H)-1,3,5-thiadiazine-2-thione (bis-THTT)

**Figure 1:**
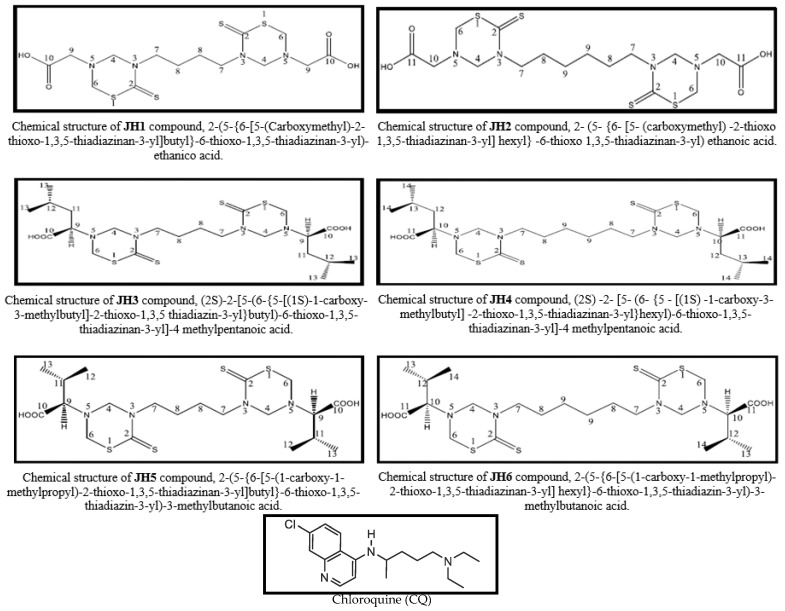
Chemical structure of bis-THTT (JH-1 to JH-6), and for Chloroquine (CQ).

This study evaluated six bis-tetrahydro-(2H)-1,3,5-thiadiazine-2-thione (bis-THTTs), labeled JH1, JH2, JH3, JH4, JH5, JH6, whose structures are shown in Figure 1, as anti-malarial compounds. Chloroquine was used as the anti-malarial drug-positive control. The bis-THTTs were previously synthesized in the Organic Synthesis Laboratory at the University of Havana and kindly donated for this research by Drs. Hortensia Rodriguez and Julieta Coro.

Bis-THTTs are comprised of two THTT heterocycles linked through the N3 positions by a lineal alkyl branch of four carbons (JH1, JH3, and JH5), six carbons (JH2, JH4, and JH6), glycine (JH1 and JH2), leucine (JH3 and JH4), and valine (JH5 and JH6) as substituents at N5 positions.

Characterization was carried out to corroborate the structures of the six bis-THTTs.

### 2.2. Characterization Techniques

The melting point (Table 1) was gauged on an Electrothermal Melting Point Apparatus. The physical property was corroborated for previously reported bis-THTTs (JH2 and JH4) [11]. FT-IR spectrometry was performed in a Cary 630 Agilent FTIR spectrometer. The analysis was carried out by the Total Reflection Attenuated (ATR) method in a wavelength range of 4000–400 cm^−1^.

The system used for UHPLC was Dionex UltiMate 3000, with a UV/Vis detector at wavelength 254 nm, and Chromeleon software 7.2.10. The injection volume was 20 µL of a 10 μg/mL sample concentration at a flow of 1 mL/min. Linear gradient elution was performed at room temperature with type 1 water and acetonitrile from 0% to 100% acetonitrile in 8 min.

### 2.3. Indirect ELISA Test Using the Experimental Compounds as Antigens

Voller’s protocol [19] was followed to obtain the indirect ELISA test’s optimal conditions. The assay was carried out in the following steps. (1) ELISA plates (96-well Nunc) were sensitized, with 100 μL of the soluble compounds in each well, with 10 μg/mL of concentration, and diluted in carbonate-bicarbonate buffer at pH 9.6 (0.5 M Na_2_CO_3_ and 0.35 M NaHCO_3_). The plates were incubated in a humid chamber at 4 °C overnight. Next, they were washed (3 times for 3 min each time) using 200 µL per well of phosphate-buffered saline and Tween 20 at 0.005% (PBS/T). (2) The wells were blocked with 100 μL of Bovine Serum Albumin (BSA) at 3%, diluted in PBS buffer. Then, the plates were incubated in a humid chamber at 37 °C for 1 h. After this time, the plates were washed (3 times for 3 min each) with PBS/T solution. (3) Next, 100 μL of PBS were added to the wells as the negative control, with hyper-immune sera of immunized BALB/c mice, along with the antigens (CQ, JH1, JH2, JH3, JH4, JH5 and JH6), diluted in PBS Buffer at 1:200. Subsequently, the plates were incubated in a humid chamber at 37 °C for 1 h. Then, the plates were washed (3 times for 3 min each time) with PBS/T solution. (4) Then 100 μL/well of the conjugate, anti-mouse IgG peroxidase antibody produced by goat conjugated to the peroxidase enzyme (HRP-peroxidase Horseradish; Sigma), in 1:1000 of dilution, were added in PBS Buffer. Next, the plates were incubated in a humid chamber at 37 °C for 1 h. Again, the plates’ washing was carried out (3 times for 3 min each time) with PBS/T (Phosphate Buffer Saline/Tween 20 at 0.005%) solution. (5) Finally, 100 μL of the chromogen ABTS (2,2′-azino-bis (3-ethylbenzthiazoline-6-sulfuric acid, Sigma) was added as substrate along with 0.05% of H_2_O_2_ at 30%, in 100 µL per well. Then, plates remained in the dark for 60 min at room temperature, and were read at 405 nm. The readings were made every 5 min to determine the optimal reading time for each compound.

### 2.4. Indirect ELISA Test with Two Combinations of Antigens

The two antigens that presented similar recognition to the hype-immune sera were chosen to combine in these indirect ELISA tests, based on the cross-reactivity reactions obtained in previous indirect ELISA tests.

This indirect ELISA experiment follows the same conditions as Voller’s protocol [19]. Combinations of two antigens were used, such as CQ + JH1, CQ + JH4, JH1 + JH3, JH1 + JH4, JH2 + JH3, JH2 + JH4, JH4 + JH3, JH5 + JH4, JH5 + JH6, JH6 + JH4, JH6 + JH3.

### 2.5. Solubility of Bis-tetrahydro-(2H)-1,3,5-thiadiazine-2-thione (bis-THTTs)

Each bis-THTT was resuspended in a minimal amount of dimethyl sulfoxide (DMSO) as the co-solvent, and then sterile, distilled water was added to arrive at 1 mg/mL (Table 1). The solutions were sonicated for 5 min to ensure complete dissolution.

### 2.6. Strains and Culture Medium

The strains used in this study to evaluate antimicrobial activity were *Staphylococcus aureus* ATCC 25923, *S. aureus* SA11-SEA (isolate from a foodborne outbreak, enterotoxin A-producing strain), *S. aureus* SA36-ORSA (clinical isolate, resistant to oxacillin), *Listeria monocytogenes* CVCM 446, *Escherichia coli* ATCC 25922, *Pseudomonas aeruginosa* ATCC 10145 and *Salmonella enteritidis* CVCM 497. The strains were reactivated in Brain Hearth Infusion, BHI (Merck, Darmstadt, Germany), and incubated at 37 °C for 18 h. The culture media used were the following: trypticase soy agar, ATS (Oxoid, Basingstoke, England) to prepare the suspensions and Muller Hinton agar, MHA (Oxoid) for the antimicrobial assay.

### 2.7. Antimicrobial Assay

The antimicrobial activity of bis-THTT was evaluated in vitro by the agar disk diffusion method [20,21]. The indicator bacteria inoculum was prepared by direct colony suspension (obtained in ATS, 35 °C for 18 h) in 0.85% NaCl (p/v) until it reached a turbidity equivalent to the 0.5 McFarland standard (~10^8^ CFU/mL). Suspensions were swabbed on MHA plates and left for 5 min at room temperature. Then, 10 µL of each of the bis-THTT and CQ compounds were dispensed into 6 mm diameter sterile filter paper discs (Wathman No. 1) and placed on the agar surface of the plates. After incubation at 35 °C for 16–18 h, the inhibition halos (mm) were measured with a vernier (Digital Caliper, Mitutoyo, Kawasaki, Japan). Assays were performed in duplicate in two independent experiments.

### 2.8. Statistical Analysis

Statistical analyses were performed with four replicates for each variable, and the program used to analyze the data was MINITAB. The first step in the study was to test for normality using the Anderson-Darling test. Data that did not present a normal distribution were transformed using the Johnson transformation. The equality of variance was determined using the Levene test (*p* > 0.05). We used a one-way ANOVA test to determine if the means were statistically significantly different (*p* < 0.05) in the cross-reactivity between the compounds, followed by a Tukey test to determine which specific samples were different.

## 3. Results

All bis-THTT were solubilized with a mixture of H_2_O/DMSO, as shown in Table 2, for BALB/c mice immunization assays and indirect ELISA.

### 3.1. Characterization of Bis-tetrahydro-2(1H)-thiadiazine-2-thiones (bis-THTT, JH1-JH6)

The structure of bis-THTTs were corroborated by melting point analysis, FT-IR and UV/Vis spectroscopy.

**Figure 2:**
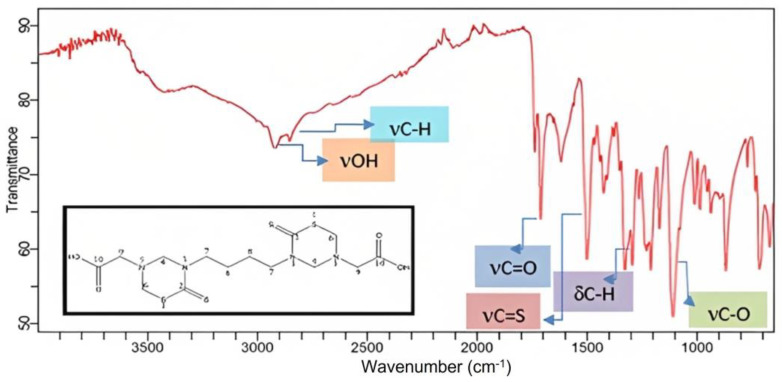
FT-IR absorption spectrum of 2-(5-{6-[5-(Carboxymethyl)-2-thioxo-1,3,5-thiadiazinan-3-yl]butyl}-6-thioxo-1,3,5-thiadiazinan-3-yl)-ethanico acid (JH1). On the X-axis are represented the wavenumber (cm^−1^) vs. transmittance on the Y-axis.

The FT-IR spectra of JH-1–JH-6 showed signs that allowed us to corroborate the unequivocal presence of the characteristic functional groups of bis-THTTs. For example, Figure 2 shows the JH-1 FT-IR spectrum, with the presence of the distinctive band of the carbonyl (νC=O, blue) and thiocarbonyl (νC=S, red) groups around 1720 cm^−1^ and 1480 cm^−1^, respectively, but also the broad characteristic band of the hydroxyl group (OH) at around 3000 cm^−1^ (νOH, orange). As additional signals, it is possible to point those corresponding to C-H stretching (νC-H) of the aliphatic chain that joins both rings through the nitrogen at position three at around 2800 cm^−1^, and the band around 1338 cm^−1^, corresponding to C-H bending of methylenes (δC-H). A strong signal at 1125 cm^−1^ corresponding to C-O stretching (νC-O) around 1125 cm^−1^ was also observed.

**Figure 3:**
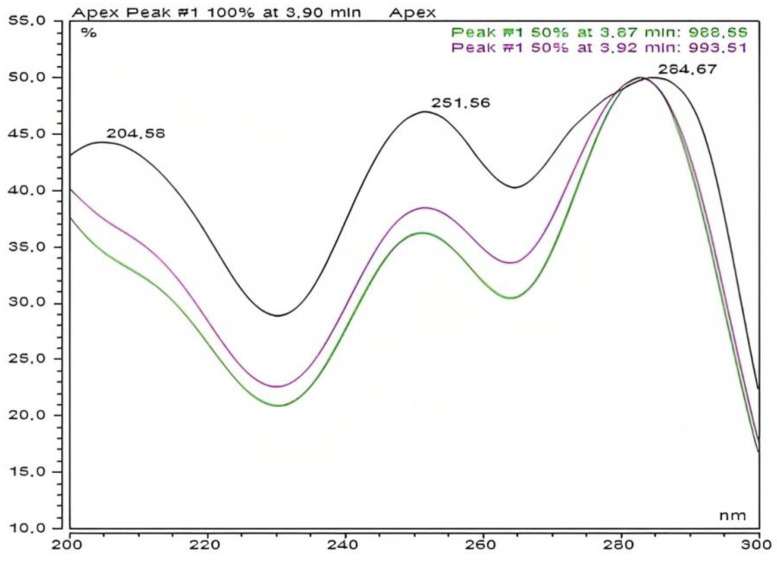
UV/Vis absorption spectrum of 2-(5-{6-[5-(Carboxymethyl)-2-thioxo-1,3,5-thiadiazinan-3-yl]butyl}-6-thioxo-1,3,5-thiadiazinan-3-yl)-ethanico acid (JH1). On the X-axis is represented the wavelength (nm) vs. absorbance on the Y-axis.

Related to UV/Vis spectroscopy, all analyzed compounds showed two characteristic bands in the UV spectra, near 290 and 250 nm (Figure 3). The intense band at 290 nm corresponded to n-π* transition of the thione group. This assignation has been confirmed by previous studies, which showed that the above mentioned signal disappears when the thiadiazine undergoes a ring cleavage [22]. The less intense band near 250 nm would be assigned to electronic transition corresponding to the carbonyl groups present in each molecule.

On the other hand, the UHPLC profiles for JH1, JH2, JH4, and JH6 showed acceptable purities between 70% to 94% (See Supplementary Materials Section). However, UHPLC profiles for JH3 and JH5 exhibited a complex chromatogram, where the signal corresponding to the bis-THTT derivative is unclear. Therefore, recrystallization of JH3 and JH5 was carried out to achieve a better purity degree.

Once the structure, purity and solubility of the bis-THTTs were validated, the biological studies related to the Indirect ELISA test, cross-reactivity, and antimicrobial activity were carried out.

### 3.2. Evaluation of the Experimental Compounds by Indirect ELISA Test

Hyperimmune serum was obtained from BALB/c mice inoculated with the bis-THTT (JH1–JH6), to determine the humoral immune response by indirect ELISA test. To standardize this test, 1:200 dilutions of sera and 10 μg/mL concentration of the antigens were used. In a previous study, Dr. Spencer’s group determined the dilution of serum and concentration [8]. However, the experiments of indirect ELISA were repeated three times until more accurate and precise results were obtained.

The reaction readings were made every 5 min, from time 0 until 60 min. If we compared the time reaction of the pre-immune (PIS) and hyper-immune sera (CQS), the best time to observe the difference between PIS and CQS was at 10 min for CQ, 15 min for JH1 and JH2, and 20 min for JH3, JH4, JH5, JH6 (data not shown).

### 3.3. Cross-Reactivity between Bis-THTT (JH1–JH6)

**Figure 4:**
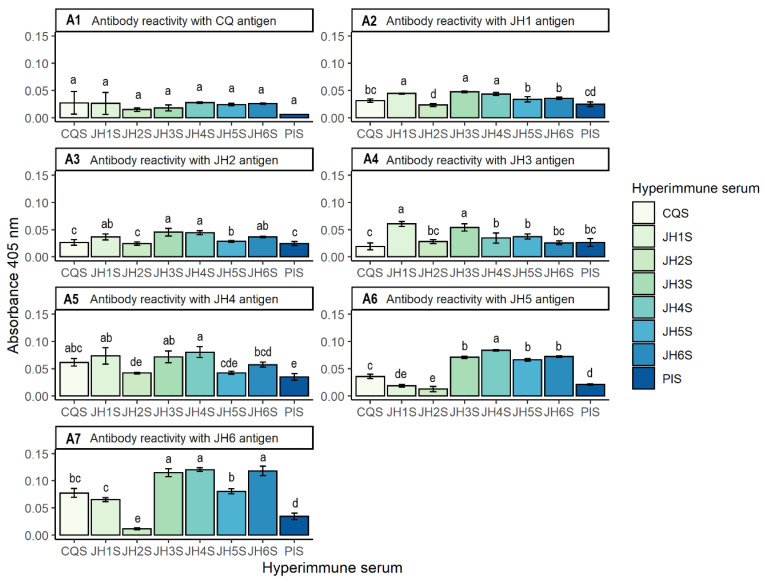
Results of indirect ELISA (Panels **A1**, **A2**, **A3**, **A4**, **A5**, **A6** and **A7**), with 10 µg/mL concentration of antigens (CQ, JH1, JH2, JH3, JH4, JH5 and JH6). On the X-axis are represented the 1:200 dilutions of the hyperimmune sera vs. absorbance at 405 nm on the Y-axis.

Figure 4 presents the cross-reactivity between bis-THTT (JH1–JH6) with pre-immune and hyperimmune serum, evaluated in the indirect ELISA test. Panel A1 shows CQ recognition results by polyclonal antibodies in hyper-immune and pre-immune sera, this last as control for reactivity in the indirect ELISA. The absorbance values were subtracted from the PBS value as a blank.

The absorbances with the different antigens are represented in the same way in panels A2, A3, A4, A5, A6 and A7 for the bis-THTT JH1, JH2, JH3, JH4, JH5 and JH6, respectively. Furthermore, the letters a, b, c, d, and e represent the significant differences in the mean absorbances between the polyclonal sera based on the statistical Tukey test. The letters (a, b) obtained in the Turkey test showed that all sera recognize JH5 as an antigen, but at different magnitudes.

The graphics of Figure 4 show the antibodies’ recognition, representing a cross-reactivity between the six bis-THTT (JH1–JH6). A black line represents CQ with hype-immune and pre-immune sera as bars. The standard error is shown above them. Furthermore, the letters a, b, c, d, and e represent the significant differences in the mean absorbances between the polyclonal sera, based on the statistical Tukey test.

Table 3 shows the cross-reactivity and the order of the absorbances obtained in the indirect ELISA experiments. The sera’s order that recognizes its antigen is highlighted. This table allows the researchers to view globally the results related to the combinations of the compounds to be tested with the hyperimmune sera in an indirect ELISA assay.

### 3.4. Evaluation of Cross-Reactivity between the Combination of Two Compounds as Antigens in Indirect ELISA

The first three compounds of each cross-reactivity obtained by each bis-THTT were selected from Table 3. In total, 11 antigen combinations resulted. An indirect ELISA test was made using antigens: CQ + JH1, CQ + JH4, JH1 + JH3, JH1 + JH4, JH2 + JH3, JH2 + JH4, JH4 + JH3, JH5 + JH4, JH5 + JH6, JH6 + JH4, JH6 + JH3, pre-immune and antigen serum in order to see the cross-reactivity, and the results are shown in Figure 4. Twenty minutes was chosen as the best reading time for all combinations of antigens.

Therefore, compound JH6 showed the highest antibody response (0.118 O.D.) followed by JH4 and JH5, with absorbances of 0.080 and 0.066, respectively. In contrast, the antigens with less humoral response stimulation are CQ, with an absorbance of 0.027, followed by JH1, with an absorbance of 0.044. Therefore, they are the least immunogenic, because they do not present a greater humoral response.

Finally, results suggest that the best combinations of two antigens as a combined therapy would be: JH1 + JH3, JH1 + JH4, JH2 + JH3, and JH2 + JH4, based on their absorbance values obtained by indirect ELISA test.

### 3.5. Antimicrobial Activity

**Figure 5:**
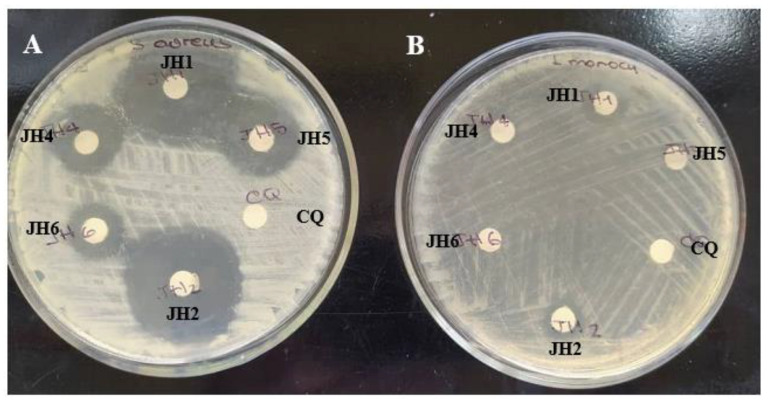
In vitro antimicrobial activity of bis-THTTs (JH1, JH2, JH4, JH5 and JH6) on Gram-positive bacteria. (**A**): *Staphylococcus aureus* ATCC 25923, (**B**): *Listeria monocytogenes* CVCM 446. CQ was used as the positive control.

**Figure 6:**
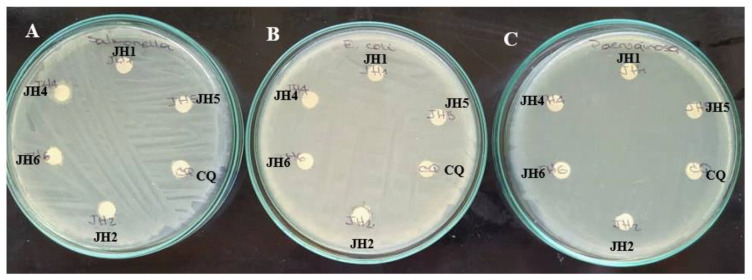
In vitro antimicrobial activity of bis-THTTs (JH1, JH2, JH4, JH5 and JH6) on Gram-negative bacteria. (**A**): *Salmonella enteritidis* CVCM 497, (**B**): *Escherichia coli* ATCC 25922, (**C**): *Pseudomonas aeruginosa* ATCC 10145. CQ was used as the positive control.

**Figure 7:**
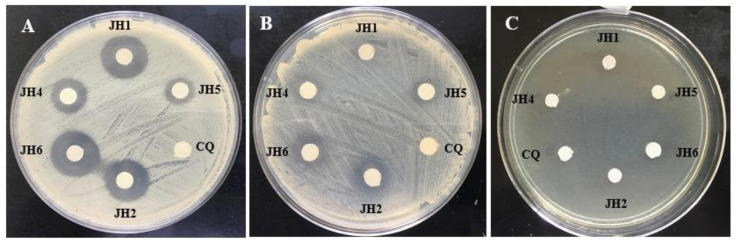
Antimicrobial activity test of bis-THTTs (JH1, JH2, JH4, JH5 and JH6) on different strains of *Staphylococcus aureus*. (**A**): *S. aureus* ATCC25923, (**B**): Enterotoxigenic *S. aureus* (SEA), (**C**): *S. aureus* ORSA. CQ was used as the positive control.

Related to antimicrobial activity the bis-THTTs, showed good inhibitory activity against Gram-positive bacteria and low or no activity against Gram-negative bacteria, as shown in Table 4 and Figure 5 and Figure 6. *S. aureus* was more susceptible to all the tested compounds [23,24]. Therefore, activity was evaluated against different *S. aureus* strains, such as *S. aureus* ATCC 25923, *S. aureus* SA11-SEA (enterotoxin A-producing strain) and *S. aureus* SA36-ORSA (oxacillin-resistant strain). Results can be seen in Table 5 and Figure 7, where the inhibitory activity was variable depending on the *S. aureus* strains. *S. aureus* ATCC 25923 and *S. aureus* SA11-SEA strains were susceptible to all compounds assayed. However, *S. aureus* SA36-ORSA strain only showed susceptibility to JH1. Therefore, JH1, JH2 and JH6 presented the most significant antimicrobial activity. In addition, JH1 and JH2, which contain glycine as a substituent in the N5 position, showed a more effective antimicrobial activity.

## 4. Discussion

Several simple or combined drugs have been used to treat Malaria, such as chloroquine [25], primaquine [26], tafenoquine [27], artemisinin [5], atevaquone-proguanil, mefloquine-artesunate [28], quinine-clindamycin [29], and artesunate-amodiaquine [30], among others. These strategies have not been enough; in consequence, the present work has studied six bis-THTTs as potential bioactive molecules to treat Malaria. In previous studies [19,22,23], the anti-parasitic activity of bis-THTTs have been evaluated against *Trypanosoma cruzi*, *T. vaginalis*, *T. b. rhodesiense* and *Leishmania donovani* [18,31,32,33]. Therefore, the humoral response by indirect ELISA test with hyperimmune sera from immunized BALB/c mice with bis-THTT were studied in terms of their cross-reaction activity by indirect ELISA [14].

Characterization techniques (melting point, UV/Vis, and FT-IR) allowed the researchers to corroborate the bis-THTT structures, assigning the signals to the most representative functional groups. On the other hand, UHPLC analysis showed that JH1, JH2, JH4, and JH6 had acceptable degree purities. In contrast, JH3 and JH5 needed to be recrystallized to achieve better purities.

The bis-THTTs have a similar structural scaffold. Therefore, these compounds are expected to show cross-reactivity between them. The results can be rationalized based on the non-polar aliphatic amino acids residue (glycine (JH1 and JH2), leucine (JH3 and JH4), and valine (JH5 and JH6)) of each bis-THTT at N5, or along the compound’s chain (four carbons (JH1, JH3 and JH5) or six carbons (JH2, JH4, and JH6).

JH1, JH3, and JH4 react with almost all polyclonal antibodies from different antigens (bis-THTT). The JH6 antigen stimulated the vertebrate BALB/c mice’s immune system and presented the highest absorbance, which was 0.118. In summary, the cross-reactivity results outlined in Figure 4 suggest that the most immunogenic compounds are JH1, JH3, and JH4, presenting four to six cross-reactions. On the other hand, the least immunogenic are Chloroquine, JH2, and JH5, and the most immunogenic compound for the BALB/c rodent model was JH6. The results suggest that the amino acid residues at N5 of the bis-THTTs are not relevant in most studied THTTs, except the compounds JH3 and JH4, which present Leucine residue at N5 and vary in the carbon chain that separates the two THTT heterocycles in each. This indicates that polyclonal antibodies recognize these two compounds (JH3 and JH4), proposing their use in combined therapies, even with JH1.

JH6 presented a high immunogenicity in indirect ELISA assay, which suggests that it is not a good drug candidate, because it is recognized as an antigen by the vertebrate’s humoral response. Furthermore, the JH3S and JH4S sera also recognize JH6 with absorbance values over 0.100, suggesting that JH6 is structurally very similar to these compounds. The difference of one more methylene (-CH_2_-) in leucine is not relevant, regarding valine, in the structure recognized by antibodies.

Finally, we suggest that the best combinations of two antigens as a combined therapy would be: JH1 + JH3, JH1 + JH4, JH2 + JH3, and JH2 + JH4. They share similar absorbances between the mixture of antigens, suggesting a similar recognition by the antibodies of both hyperimmune sera between both compounds combined, which could be used in combination therapy in a murine malaria model.

There are reports in the literature that evaluate the cross-reactivity between antigens using the indirect ELISA test with anti-malarial drugs [34,35,36,37]. Therefore, our results related to a cross-reactivity experiment carried out with antibodies (hyperimmune sera) obtained from BALB/c mice with several anti-malarial activities.

Bis-THTTs showed good antimicrobial activity against Gram-positive bacteria and low or no activity against Gram-negative bacteria. Similar results have been reported in the evaluation of thiadiazine derivatives [23,27,38,39,40]. All bis-THTTs showed good activity against *S. aureus* ATCC 25923 and SA11-SEA strains, but not against *S. aureus* SA36 (resistant to multiple antibiotics strains), so the susceptibility was variable between strains [40]. The compounds JH1, JH2 and JH6 showed evident halos of inhibition in the growth of *S. aureus* ATCC 25923. However, JH1 and JH2 were the most effective in their antimicrobial activity, suggesting that biological activity could depend on the compound’s chemical structure [41]. Changes in the substituent amino acid residues at the N5 position of the thiadiazine nucleus influenced increasing antimicrobial activity. These results agree with the evaluation of related thiadiazine derivates [41,42,43,44].

Accordingly, compound JH6 showed the highest antibody response (0.118 O.D.), followed by JH4 and JH5 with absorbances of 0.080 and 0.066, respectively. In contrast, the antigens with less humoral response stimulation are CQ, with an absorbance of 0.027, followed by JH1, with an absorbance of 0.044. Consequently, they are the least immunogenic because they do not present a greater humoral response.

## 5. Conclusions

The structure and purity of six bis-THTTs (JH1-JH6) were corroborated by melting point (mp), FT-IR, UV/Vis spectroscopy, and UHPLC, allowing their use as antigens in the generation of hyperimmune sera obtained from immunized BALB/c mice.

The bis-THTTs (JH1–JH6) have a similar structural scaffold. Therefore, the cross-reactivity evaluation was a good and accessible strategy for determining the analysis by antibody recognition. The humoral evaluation results through indirect ELISA show that compounds JH1, JH3, and JH4 react with almost all of the compounds, or are recognized by the rest of the compounds’ polyclonal antibodies. The JH4, JH5, and JH6 antigens stimulated the BALB/c mice’s immune system and presented the highest absorbances.

The cross-reactivity experiment demonstrated that the combinations JH1 + JH3, JH1 + JH4, JH2 + JH3, and JH2 + JH4 could be a combined therapy, because they share absorbances between the mixture of antigens. These suggest a recognition by antibodies showing a similar structure as epitopes in the Fab region by compounds.

Our results show that the indirect ELISA test is a good tool for evaluating the cross-reactivity between bis-THTTs, allowing us to suggest combinations between them for a future application as antiprotozoals.

Finally, the different bis-THTTs showed antimicrobial activity on Gram-positive bacteria, mainly on strains of *S. aureus* ATCC 25923 and SA11 (SEA), the compounds JH1 and JH2 being more effective. No inhibitory activity was observed on the tested Gram-negative bacteria.

## Figures and Tables

**Table 1:** Melting point of bis-THTTs (JH1–JH6).

Compound	Melting Point (°C)
JH1	130–132
JH2	134–136 (134–136) *
JH3	132–134
JH4	96–98 (95–97) *
JH5	121–124
JH6	131–133

* Melting points reported in [18].

**Table 2:** Preparation of bis-THTT solutions.

Diluents (µL)	JH1	JH2	JH4	JH5	JH6	CQ
Distilled water	920	940	810	800	750	970
DMSO	80	60	190	200	250	30

**Table 3:** Cross-reactivity of antibody with antigen from highest to lowest humoral response. Each antigen’s order of cross-reactivity is presented, and each hyperimmune sera is highlighted in bold.

Experimental Compounds	Sequence of Cross Reactivity Reponse
CQ	**CQS** > JH4S > JH1S > JH6
JH1	JH3S > **JH1S** > JH4S
JH2	JH3S > JH4S > **JH2S**
JH3	JH1S > **JH3S** > JH5S
JH4	**JH4S** > JH1S > JH3S > CQS
JH5	JH4S > JH6S > JH3S > **JH5S**
JH6	JH4S > **JH6S** > JH3S

**Table 4:** Antimicrobial activity of bis-THTT’ ‘s (JH1, JH2, JH4, JH5 and JH6) on Gram-positive and Gram-negative bacteria.

Indicator Bacteria ^(a)^	Thiadiazine Derivatives ^(b)^				
	JH1	JH2	JH4	JH5	JH6
*Escherichia coli* ATCC 25922	-	8.25 ± 0.31	-	-	-
*Listeria monocytogenes* CVCM 446	12.78 ± 0.72	10.45 ± 0.42	8.56 ± 0.20	-	-
*Salmonella enteritidis* CVCM 497	-	-	-	-	-
*Staphylococcus aureus* ATCC 25923	32.32 ± 1.23	32.37 ± 0.62	19.87 ± 1.00	21.81 ± 0.46	16.03 ± 0.65
*Pseudomonas aeruginosa* ATCC 10145	-	-	-	-	-

^a^ Abbreviation: ATCC, American Type Culture Collection (Rockville, MD, USA); CVCM, Venezuelan Center Collection of Microorganisms (Caracas, Dept. Capital, Venezuela); ^b^ Inhibition diameter (mm ± SD).

**Table 5:** Antimicrobial activity of bis-THTTs (JH1, JH2, JH4, JH5 and JH6) on different strains of *Staphylococcus aureus*.

Strain	Origin ^(a)^	Thiadiazine Derivates ^(b)^				
		JH1	JH2	JH4	JH5	JH6
*S. aureus* ATCC 25923	ATCC	17.57 ± 0.61	18.45 ± 1.40	14.83 ± 0.49	11.75 ± 0.35	19.74 ± 0.49
*S. aureus* SA11 (SEA)	CL	9.10 ± 0.39	12.31 ± 0.05	9.50 ± 0.36	9.21 ± 0.66	11.94 ± 0.20
*S. aureus* SA36 (ORSA)	CL	11.38 ± 0.28	-	-	-	-

^a^ Abbreviation: ATCC, American Type Culture Collection (Rockville, MD, USA); CL, Collection of Microbiology Laboratory, Dept. of Technology of Biological and Biochemical Processes, Simón Bolivar University (Caracas, Miranda, Venezuela) ; SA11 (SEA): enterotoxin A-producing enterotoxigenic strain isolated from a food outbreak; SA36 (ORSA) oxacillin resistant strain, clinical isolate. ^b^ Inhibition diameter (mm ± SD).

## Data Availability

Not applicable.

## Supplementary Materials

The following supporting information can be downloaded at: https://www.mdpi.com/article/10.3390/mps6020037/s1.
