# Supplementary material for: Serological Cross-Reaction between Six Thiadiazine by Indirect ELISA Test and Their Antimicrobial Activity
Source: Methods Protoc. 2023 Apr 3;6(2):37. doi: 10.3390/mps6020037 (PMC10146945; doi:10.3390/mps6020037)
Supplement: Supplementary file 1 [file mps-06-00037-s001.zip › mps-2147689-supplementary.pdf]

Serological cross-reaction between six Thiadiazine by indirect ELISA test and their anti-microbial activity.

Mishell Ortiz[1], Hortensia Rodriguez[2], Elisabetta Lucci[3], Julieta Coro[4], Beatriz Pernia[5], Abigail Montero[1], Francisco Javier Tingo-Jácome[7], Leslie Espinoza[1] and Lilian Spencer[1,6*]

[1]School of Biological Sciences and Engineering, Yachay Tech University, San Miguel de Urcuquí, Ecuador; https://orcid.org/0000-0001-7783-620X; mishell.ortiz@yachaytech.edu.ec; lspencer@yachaytech.edu.ec; spencerlilian@gmail.com

[2]School of Chemical Sciences and Engineering, Yachay Tech University, San Miguel de Urcuquí, Ecuador; hmrodriguez@yachaytech.edu.ec

[3]Dpto. de Tecnología de Procesos Biológicos y Bioquímicos. Universidad Simón Bolívar, Venezuela, elucci@usb.ve

[4]Laboratory of Organic Synthesis, Faculty of Chemistry, Habana University, 10400 Habana, Cuba.

[5]University of Guayaquil, Faculty of Natural Sciences, Av. Raúl Gómez Lince s/n y Av. Juan Tanca Marengo, Guayaquil, Ecuador. https://orcid.org/0000-0002-2476-7279; beatrizpernia@gmail.com

[6]Cell Biology Department, Simón Bolívar University, Valle de Sartenejas, Caracas, Venezuela

[7]Biology Center, Central University of Ecuador, Quito, Ecuador.

* Correspondence should be addressed to Lilian M. Spencer; spencerlilian@gmail.com

Supporting Information

Result of UHPLC

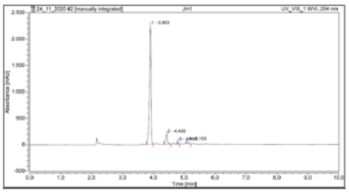

(a)UHPLC profile of JH1 compound, 2-(5-{6-[5-(Carboxymethyl)-2-thioxo-1,3,5-thiadiazinan-3-yl]butyl}-6-thioxo-1,3,5-thiadiazinan-3-yl)-ethanico acid. (b)UHPLC profile of acetonitrile blank

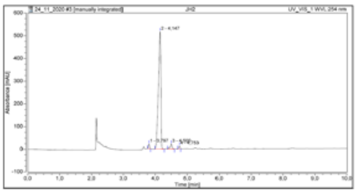

(a)UHPLC profile of JH2 compound, 2- (5- {6- [5-(carboxymethyl) -2-thioxo 1,3,5-thiadiazinan-3-yl] hexyl} -6-thioxo 1,3,5-thiadiazinan-3-yl) ethanoic acid. (b)UHPLC profile of acetonitrile blank

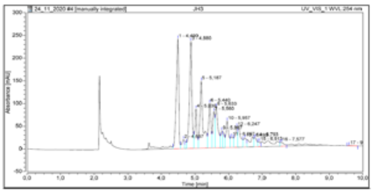

(a)UHPLC profile of JH3 compound, (2S)-2-[5-(6-{5-[(1S)-1-carboxy-3-methylbutyl]-2-thioxo-1,3,5 thiadiazin-3-yl}butyl)-6-thioxo-1,3,5-thiadiazinan-3-yl]-4 methylpentanoic acid. (b)UHPLC profile of acetonitrile blank

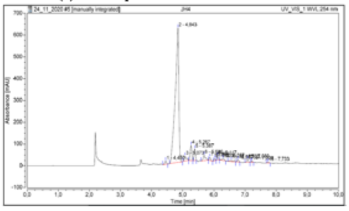

(a)UHPLC profile of JH4 compound, (2S) -2- [5- (6- {5 -[(1S) -1-carboxy-3-methylbutyl] -2-thioxo-1,3,5-thiadiazinan-3-yl}hexyl)-6-thioxo-1,3,5-thiadiazinan-3-yl]-4 methylpentanoic acid. (b)UHPLC profile of acetonitrile blank

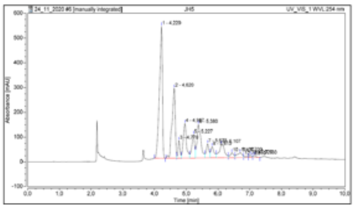

(a)UHPLC profile of JH5 compound, 2-(5-{6-[5-(1-carboxy-1-methylpropyl)-2-thioxo-1,3,5-thiadiazinan-3-yl]butyl}-6-thioxo-1,3,5-thiadiazin-3-yl)-3-methylbutanoic acid. (b)UHPLC profile of acetonitrile blank

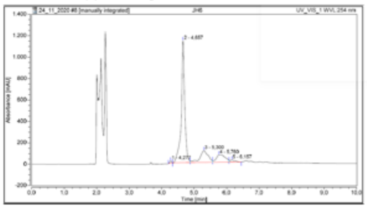

(a)UHPLC profile of JH6 compound, 2-(5-{6-[5-(1-carboxy-1-methylpropyl)-2-thioxo-1,3,5-thiadiazinan-3-yl] hexyl}-6-thioxo-1,3,5-thiadiazin-3-yl)-3-methylbutanoic acid. (b)UHPLC profile of acetonitrile blank
